# Decreases in self-reported alcohol consumption following HIV counseling and testing at Mulago Hospital, Kampala, Uganda

**DOI:** 10.1186/1471-2334-14-403

**Published:** 2014-07-20

**Authors:** Judith A Hahn, Robin Fatch, Rhoda K Wanyenze, Steven Baveewo, Moses R Kamya, David R Bangsberg, Thomas J Coates

**Affiliations:** 1 University of California, San Francisco, Box 0886, San Francisco, CA 94143-0886, USA; 2 Makerere University, Kampala, Uganda; 3 Marie Stopes Uganda, Kampala, Uganda; 4 Massachusetts General Hospital, Boston, MA, USA; 5 University of California, Los Angeles, USA

**Keywords:** Alcohol, Africa, HIV, HIV counseling and testing, Antiretroviral therapy, Screening and brief intervention

## Abstract

**Background:**

Alcohol use has a detrimental impact on the HIV epidemic, especially in sub-Saharan Africa. HIV counseling and testing (HCT) may provide a contact opportunity to intervene with hazardous alcohol use; however, little is known about how alcohol consumption changes following HCT.

**Methods:**

We utilized data from 2056 participants of a randomized controlled trial comparing two methods of HCT and subsequent linkage to HIV care conducted at Mulago Hospital in Kampala, Uganda. Those who had not previously tested positive for HIV and whose last HIV test was at least one year in the past were eligible. Participants were asked at baseline when they last consumed alcohol, and prior three month alcohol consumption was measured using the Alcohol Use Disorders Identification Test – Consumption (AUDIT-C) at baseline and quarterly for one year. Hazardous alcohol consumption was defined as scoring ≥3 or ≥4 for women and men, respectively. We examined correlates of alcohol use at baseline, and of hazardous and non-hazardous drinking during the year of follow-up using multinomial logistic regression, clustered at the participant level to account for repeated measurements.

**Results:**

Prior to HCT, 30% were current drinkers (prior three months), 27% were past drinkers (>3 months ago), and 44% were lifetime abstainers. One-third (35%) of the current drinkers met criteria for hazardous drinking. Hazardous and non-hazardous self-reported alcohol consumption declined after HCT, with 16% of baseline current drinkers reporting hazardous alcohol use 3 months after HCT. Independent predictors (p < 0.05) of continuing non-hazardous and hazardous alcohol consumption after HCT were sex (male), alcohol consumption prior to HCT (hazardous), and HIV status (negative). Among those with HIV, non-hazardous drinking was less likely among those taking antiretroviral therapy (ART).

**Conclusions:**

HCT may be an opportune time to intervene with alcohol consumption. Those with HIV experienced greater declines in alcohol consumption after HCT, and non-hazardous drinking decreased for those with HIV initiating ART. HCT and ART initiation may be ideal times to intervene with alcohol consumption. Screening and brief intervention (SBI) to reduce alcohol consumption should be considered for HCT and HIV treatment venues.

## Background

Heavy alcohol consumption is known to have detrimental effects on health, accounting for approximately 4.5% of the global burden of disease and injury world-wide [1]. Heavy alcohol use is a common and growing problem in sub-Saharan Africa (SSA). In Uganda, heavy alcohol consumption among drinkers is especially common [1]. Among male drinkers, the per capita yearly pure alcohol consumption is 25.6 liters; among female drinkers, the per capita yearly pure alcohol consumption is 19.6 liters [1].

Sub-Saharan Africa is home to nearly 70% of the global HIV infections (UNAIDS report 2011), and the heavy alcohol consumption in this region exacerbates the problem for multiple reasons [2]. First, alcohol consumption and drinking venue attendance in SSA have been associated with increased HIV risk behaviors, such as number of sexual partners, unprotected sex, and commercial sex work [3,4], as well as prevalent [5,6] and incident HIV infection [7-9]. Alcohol consumption has also been associated with decreased access to HIV testing [10]. In addition, several studies of alcohol administration to macaques demonstrated increased SIV disease progression [11]; however, observational studies of heavy alcohol consumption and HIV disease progression in humans have yielded mixed results [12-14]. Finally, alcohol consumption is consistently associated with decreased antiretroviral therapy (ART) adherence in western countries [15], and increasingly in SSA [16-23], and heavy alcohol consumption has been associated with decreased retention in care [24].

We recently found that self-reported alcohol consumption by persons with HIV decreased concurrently with ART initiation in rural Uganda; almost two-thirds (64%) of drinkers at baseline reported becoming and remaining abstinent for the duration of follow-up, for a median of 3.25 years [25]. Most became abstinent within three months of starting ART, suggesting that ART initiation may be an important time to intervene to reduce alcohol consumption. Reductions in alcohol use among women in a multicenter HIV cohort study in the United States have also been reported [26]. However, changes in alcohol use earlier in the course of HIV care, that is, after HIV counseling and testing (HCT), have not been examined. Because alcohol appears to play an important role in HIV transmission, reducing alcohol consumption early in the course of HIV could have an important impact on the HIV epidemic. HCT provides a point of health care contact that might be an opportune time to intervene on heavy alcohol consumption [27]. Current standard HCT guidelines, e.g. those published by the United States Centers for Disease Control and Prevention [28], recommend addressing the use of alcohol or drugs before sexual activity, but alcohol use itself is not directly addressed. Declines in sexual risk behavior due to HCT have been demonstrated [29], and it is plausible that other general health improvements might additionally occur after HCT.

For the above reasons, we sought to examine changes in alcohol use in the year following HCT. We utilized data collected in a randomized controlled trial of HCT methods and enhanced linkage to HIV care at Mulago Hospital in Kampala, Uganda [30] to examine whether alcohol use declined in the year following HCT. We examined whether changes in alcohol consumption differed by HIV status and sex, and examined demographic and other factors as predictors of hazardous and non-hazardous drinking following HCT. Among those with HIV, we examined whether CD4 cell count at baseline and initiation of ART were associated with declines in drinking after HCT.

## Methods

This study is an analysis of data collected as part of a randomized controlled trial comparing an abbreviated method of HCT to traditional full length HCT, and, among those with HIV, comparing an enhanced protocol for linkage to HIV care to standard linkage to care. Study details, including a detailed flow diagram, have been presented elsewhere [30].

### Study population

Participants were recruited from May 2008 to June 2011 from inpatient medical wards and outpatient clinics (including emergency and casualty wards, medical outpatient clinics, and sexually transmitted disease clinics) at Mulago Hospital in Kampala, Uganda. Eligibility criteria included being ≥18 years old, residing within 25 km of Mulago Hospital with no plans of moving, having never tested positive for HIV, having last tested HIV-negative at least one year prior, and being willing to receive an HIV test and engage in study procedures. All participants provided informed consent prior to study participation and the study was approved by the institutional review boards of Makerere University, Uganda National Council for Science and Technology, University of California Los Angeles, and University of California San Francisco.

### Study visits and procedures

All participants completed a 30-minute, interviewer-administered survey at baseline. Participants were then randomly assigned (1:1) to receive either traditional HCT (45 minutes), or an abbreviated (15 minutes) version of HCT. Following HCT and determination of HIV status, participants were randomized a second time. To retain a roughly equivalent number of HIV-positive and HIV-negative participants followed for one year, HIV-negative participants were randomized to follow-up or no follow-up (roughly 1:1). HIV-positive participants were randomized to receive enhanced or standard linkage to HIV care (1:1); all were followed for one year. Follow-up study interviews were conducted quarterly for one year, and included assessment of HIV care and ART status, alcohol consumption, and sexual behaviors within the past three months.

### Dependent variables

Alcohol use prior to baseline was examined using four categories: lifetime abstainer, past drinker (drank >3 months prior), current (prior three months) non-hazardous drinker, and current (prior three months) hazardous drinker. We defined hazardous drinking using the Alcohol Use Disorders Identification Test – Consumption (AUDIT-C) [31] with a cut-off of ≥3 for women and ≥4 for men; non-hazardous drinking was defined as any current drinking that did not reach the AUDIT-C cut-off. Following HCT, drinking in the prior three months among those reporting current drinking at baseline was examined as: none, non-hazardous drinking and hazardous drinking, as defined above.

### Covariates

Covariates of interest included demographics: participant sex, age, marital status, education, occupation, and religion. We created a household wealth index to group households based on ownership of durable goods, housing quality, and energy sources [32]. This variable was divided into three categories: low (0-40%), middle (41-80%), and high (81-100%) household wealth. We asked about frequency of household hunger: sometimes/often (>2 times/month), seldom (1–2 times/month), or never. We included site of recruitment (inpatient ward, outpatient clinic, or emergency/casualty ward) as a potential proxy for participant health. We examined social support using the Oslo Social Support scale [33], and categorized participants as having poor (score of 3–8), moderate (9–11), or strong (12–14) social support. We also examined whether any household members consumed alcohol in the past three months. Reason(s) for HIV testing were also examined; while the questionnaire allowed a participant to endorse more than one reason, we created a hierarchical variable from these reasons: (a) concerned about health/symptoms, (b) wanted to plan for the future or just wanted to know one’s status, and (c) other reasons. Among those with HIV, ART status was ascertained using a list of medications at each follow-up interview. We also included HCT study arm (traditional or abbreviated) and, among those with HIV, linkage to HIV care study arm (enhanced or standard), in the analyses of alcohol use in the year following HCT.

### Laboratory testing

HIV testing was conducted using a serial testing procedure using rapid antibody tests as described previously [34]. HIV testing was conducted at Mulago Hospital; CD4 cell count testing for those with HIV infection was conducted at the Makerere University-Johns Hopkins University laboratory.

### Statistical analysis

We calculated frequency distributions for categorical variables, and medians and interquartile ranges (IQR) for continuous variables. We conducted unadjusted and adjusted multinomial logistic regression analyses for the baseline outcomes (lifetime abstainer, past drinker, current non-hazardous drinker, and current hazardous drinker). Among those who were current drinkers at baseline, we also conducted unadjusted and adjusted multinomial logistic regression analyses of alcohol use in the one year following HCT, clustering on the participant to account for the repeated measures over time. We included the study arms (HCT arm and, among those with HIV, HIV linkage to care arm) as covariates only in the analyses of alcohol use in the year following HCT, as study randomization occurred after the baseline interview. The levels of the outcome variable were: no alcohol use in the past three months, current non-hazardous use, and current hazardous use. For each of these models, we used a purposeful selection technique [35] to create multivariable models. Covariates were initially included if they were associated in bivariate models with a p-value ≤ 0.25; they were then excluded in a backwards stepwise fashion, keeping variables in the model if they were associated at p ≤ 0.10. Next, any covariates initially excluded based on the cut-off of p ≤ 0.25 were included one by one, and their significance was re-assessed. They were retained in the final model if p ≤ 0.10.

Next, to determine whether the time trends varied by HIV status or sex in our model of alcohol consumption following HCT, we conducted tests of interaction of those two variables with time in the multivariable models, one at a time, while adjusting for the other variables in the model [36]. As there was a significant interaction with HIV status and time (p = 0.02), and because we were interested in examining variables relevant only to HIV-positive participants, we also fit a multivariable model among only the participants with HIV, using the purposeful selection methods described above, and additionally allowing baseline CD4 cell count, linkage to care study arm, and ART use to enter the model.

Data were missing at baseline and follow-up (15% of observations had at least one missing value; 13% of follow-up study visits were missing), so we conducted multiple imputation using chained equations. The results using the imputed data were similar to the results using listwise deletion; therefore we present the results using the imputed data for the regression analyses.

## Results

### Participant characteristics

3389 participants were enrolled in the main study; HIV prevalence was 30% (n = 1003). 1323 HIV negatives were randomized to no follow-up, and 10 participants were missing data on baseline alcohol use; therefore 2056 participants were included in the baseline analysis. More than half of the participants (57%) were female (Table 1), median age was 30 years (IQR: 25–38), and approximately half (49%) had more than a primary education. Approximately one-third (32%) of participants were Catholic, 31% were Protestant, 19% were Moslem, and 18% were Saved, Pentecostal, or another religion. Eighteen percent (18%) of participants reported having at least one household member who had consumed alcohol in the past three months. Most participants reported that their household never went hungry (76%) and that they had moderate or strong social support (81%). The median CD4 cell count among those who were infected with HIV was 285 cells/mm^3^ (IQR: 132–463).

**Table 1:** Baseline demographic and behavioral characteristics of participants undergoing HCT in Kampala, Uganda

		**Drinking status at baseline**			
	**Overall (n = 2056)**	**Lifetime abstainers (n = 902)**	**Past drinkers (n = 547)**	Current*, non-hazardous drinkers (prior 3 months) (n = 352)	Current*, hazardous drinkers (prior 3 months) (n = 189)
	**n (%)**	**n (%)**	**n (%)**	**n (%)**	**n (%)**
**Gender**					
Male	879 (42.8)	351 (38.9)	222 (40.6)	165 (46.9)	108 (57.1)
Female	1177 (57.3)	551 (61.1)	325 (59.4)	187 (53.1)	81 (42.9)
**Age (overall median: 30; IQR: 25–38)**					
18–25	580 (28.2)	321 (35.6)	130 (23.8)	86 (24.4)	26 (13.8)
26–35	852 (41.4)	353 (39.1)	212 (38.8)	162 (46.0)	94 (49.7)
>35	624 (30.4)	228 (25.3)	205 (37.5)	104 (29.6)	69 (36.5)
**Education**					
Primary education or less	1043 (50.8)	439 (48.7)	278 (50.9)	191 (54.3)	102 (54.0)
More than primary education	1011 (49.2)	462 (51.3)	268 (49.1)	161 (45.7)	87 (46.0)
**Occupation**					
Laborer	668 (32.5)	285 (31.6)	186 (34.0)	119 (33.8)	54 (28.6)
Business/Sales/Technical	901 (43.8)	388 (43.1)	240 (43.9)	151 (42.9)	91 (48.2)
Other	486 (23.7)	228 (25.3)	121 (22.1)	82 (23.3)	44 (23.3)
**Religion**					
Protestant	629 (30.6)	217 (24.1)	161 (29.4)	135 (38.4)	88 (46.6)
Catholic	651 (31.7)	198 (22.0)	176 (32.2)	169 (48.0)	76 (40.2)
Moslem	398 (19.4)	283 (31.4)	64 (11.7)	30 (8.5)	17 (9.0)
Saved/Pentecostal/Other	378 (18.4)	204 (22.6)	146 (26.7)	18 (5.1)	8 (4.2)
**Marital status**					
Married	866 (42.1)	370 (41.0)	212 (38.8)	159 (45.2)	92 (48.7)
Married in the past	656 (31.9)	248 (27.5)	208 (38.1)	116 (33.0)	65 (34.4)
Never married	533 (25.9)	284 (31.5)	126 (23.1)	77 (21.9)	32 (16.9)
**Household wealth**					
Low	921 (45.0)	411 (45.7)	246 (45.2)	144 (40.9)	96 (50.8)
Medium	807 (39.4)	346 (38.4)	207 (38.1)	155 (44.0)	72 (38.1)
High	321 (15.7)	143 (15.9)	91 (16.7)	53 (15.1)	21 (11.1)
**How often do household members go hungry?**					
Sometimes/Often (>2 times/month)	295 (14.6)	103 (11.6)	108 (20.1)	30 (8.6)	34 (18.7)
Seldom (1–2 times/month)	200 (9.9)	66 (7.5)	61 (11.4)	39 (11.2)	28 (15.4)
Never	1522 (75.5)	717 (80.9)	368 (68.5)	280 (80.2)	120 (65.9)
**Social support**					
Strong support	274 (13.9)	107 (12.2)	81 (15.8)	36 (10.4)	35 (19.7)
Moderate support	1311 (66.7)	628 (71.9)	325 (63.4)	254 (73.6)	86 (48.3)
Poor support	380 (19.3)	139 (15.9)	107 (20.9)	55 (15.9)	57 (32.0)
**Recruitment site**					
Inpatient wards	400 (19.5)	181 (20.1)	106 (19.4)	72 (20.5)	33 (17.5)
Outpatient clinics	1395 (67.9)	608 (67.4)	378 (69.1)	222 (63.1)	138 (73.0)
Emergency/casualty wards	261 (12.7)	113 (12.5)	63 (11.5)	58 (16.5)	18 (9.5)
**Reasons for HIV testing (illness > planning > other)**					
AIDS symptoms/concern about current illness	652 (31.7)	241 (26.7)	201 (36.8)	117 (33.2)	70 (37.0)
Just wanted to know/plan for future	1312 (63.8)	624 (69.2)	320 (58.5)	225 (63.9)	105 (55.6)
Other	92 (4.5)	37 (4.1)	26 (4.8)	10 (2.8)	14 (7.4)
**Any household members consumed alcohol, past 3 months**					
None	1671 (82.0)	805 (89.4)	439 (81.5)	276 (79.5)	108 (57.8)
Any	367 (18.0)	95 (10.6)	100 (15.6)	71 (20.5)	79 (42.3)
**Last time consumed alcohol**					
Never	902 (43.9)	902 (100.0)	0 (0.0)	0 (0.0)	0 (0.0)
>5 years ago	191 (9.3)	0 (0.0)	191 (34.9)	0 (0.0)	0 (0.0)
1–5 years ago	141 (6.9)	0 (0.0)	141 (25.8)	0 (0.0)	0 (0.0)
3 months – 1 year ago	215 (10.5)	0 (0.0)	215 (39.3)	0 (0.0)	0 (0.0)
Prior 3 months	607 (29.5)	0 (0.0)	0 (0.0)	352 (100.0)	189 (100.0)
**AUDIT-C at baseline (median (IQR))**	0 (0–1)				
**HIV status**					
HIV Negative	1058 (51.5)	529 (58.7)	250 (45.7)	176 (50.0)	71 (37.6)
HIV Positive	998 (48.5)	373 (41.4)	297 (54.3)	176 (50.0)	118 (62.4)
**Baseline CD4 cell count among those with HIV (cells/mm**^**3**^**) (overall median: 285; IQR: 132–463)**					
<200	359 (36.1)	139 (37.3)	122 (41.4)	53 (30.1)	35 (29.9)
200–349	233 (23.4)	85 (22.8)	66 (22.4)	44 (25.0)	29 (24.8)
350–499	202 (20.3)	74 (19.8)	60 (20.3)	34 (19.3)	26 (22.2)
> = 500	200 (20.1)	75 (20.1)	47 (15.9)	45 (25.6)	27 (23.1)

*n = 66 current drinkers were unable to be classified as hazardous or non-hazardous drinkers.

### Baseline alcohol use

Approximately 56% of the sample had ever consumed any alcohol. Of the 1154 participants who reported ever taking alcohol, 607 (53%) reported taking alcohol in the prior three months. Among former drinkers, alcohol was last consumed 3–12 months ago for 215 participants (39%). Among current (prior three months) drinkers, 40% of men and 30% of women reported hazardous alcohol use, as defined by the AUDIT-C.

Table 2 shows the results from multinomial logistic regression of past, current non-hazardous, and current hazardous alcohol use compared to lifetime abstention. Independent correlates (p < 0.05) of past alcohol use versus lifetime abstention were: age (>35 years), religion (Protestants more likely than Moslems), marital status (previously married more likely than currently married), household hunger (never hungry less likely than sometimes/often hungry), any alcohol use by household members, and HIV status (positive). Independent correlates of current, non-hazardous alcohol use versus lifetime abstention were: sex (male), age (26–35 years), religion (Catholics more likely than Protestants; Moslems and Saved/Pentecostal/others less likely than Protestants), household wealth (medium household wealth more likely than low), and any alcohol use by household members. Lastly, independent predictors of current, hazardous alcohol consumption compared to lifetime abstention were: sex (male), age (older), religion (Moslems and Saved/Pentecostal/others less likely than Protestants), social support (strong support more likely than moderate support), any alcohol use by household members, recruitment at an outpatient clinic (versus an inpatient ward), and HIV status (positive). The relative risk ratios (RRR) increased in size across the categories from past drinking, to current non-hazardous drinking, to current hazardous drinking for sex, age, and having a household member who consumes alcohol.

**Table 2:** Multinomial logistic regression of past, current non-hazardous, and current hazardous drinking status at baseline compared to lifetime abstaining

	**Past drinking RRR (95% CI)**		**Current, non-hazardous drinking RRR (95% CI)**		**Current, hazardous drinking RRR (95% CI)**	
	**Bivariable**	**Multivariable**	**Bivariable**	**Multivariable**	**Bivariable**	**Multivariable**
**Gender**						
Female	1.00	1.00	1.00	1.00	1.00	1.00
Male	1.07 (0.86, 1.33)	1.25 (0.98, 1.60)	1.40 (1.10, 1.77)	1.57 (1.19, 2.07)	2.10 (1.52, 2.89)	2.86 (1.96, 4.16)
**Age (years)**						
18–25	1.00	1.00	1.00	1.00	1.00	1.00
26–35	1.48 (1.14, 1.93)	1.28 (0.95, 1.73)	1.69 (1.27, 2.25)	1.42 (1.01, 1.99)	3.23 (2.05, 5.08)	2.62 (1.53, 4.50)
>35	2.22 (1.68, 2.93)	1.94 (1.39, 2.71)	1.66 (1.21, 2.27)	1.33 (0.89, 1.98)	3.62 (2.25, 5.84)	2.83 (1.55, 5.18)
**Education**						
Primary education or less	1.00	-	1.00	-	1.00	-
More than primary education	0.92 (0.74, 1.13)		0.82 (0.65, 1.05)		0.81 (0.59, 1.12)	
**Occupation**						
Laborer	1.00	-	1.00	-	1.00	-
Business/sales/technical	0.95 (0.74, 1.21)		0.93 (0.71, 1.22)		1.24 (0.86, 1.79)	
Other	0.81 (0.61, 1.08)		0.81 (0.59, 1.12)		1.01 (0.65, 1.55)	
**Religion**						
Protestant	1.00	1.00	1.00	1.00	1.00	1.00
Catholic	1.20 (0.90, 1.60)	1.19 (0.88, 1.60)	1.39 (1.04, 1.85)	1.35 (1.01, 1.81)	0.92 (0.64, 1.32)	0.84 (0.57, 1.24)
Moslem	0.30 (0.22, 0.43)	0.32 (0.23, 0.45)	0.16 (0.11, 0.25)	0.17 (0.11, 0.26)	0.14 (0.08, 0.24)	0.16 (0.09, 0.30)
Saved/Pentecostal/Other	0.96 (0.72, 1.29)	1.08 (0.79, 1.47)	0.14 (0.08, 0.23)	0.15 (0.09, 0.24)	0.09 (0.04, 0.19)	0.12 (0.05, 0.25)
**Marital status**						
Married	1.00	1.00	1.00	1.00	1.00	1.00
Previously married	1.47 (1.14, 1.88)	1.44 (1.09, 1.90)	1.05 (0.80, 1.39)	1.19 (0.87, 1.64)	1.05 (0.73, 1.49)	1.51 (1.00, 2.28)
Never married	0.77 (0.59, 1.01)	0.98 (0.72, 1.35)	0.63 (0.47, 0.85)	0.72 (0.50, 1.04)	0.44 (0.28, 0.68)	0.83 (0.48, 1.44)
**Household assets**						
Low	1.00	1.00	1.00	1.00	1.00	1.00
Medium	1.00 (0.79, 1.26)	1.18 (0.91, 1.52)	1.30 (1.01, 1.69)	1.48 (1.11, 1.96)	0.89 (0.64, 1.24)	1.22 (0.84, 1.78)
High	1.06 (0.78, 1.44)	1.21 (0.87, 1.68)	1.13 (0.80, 1.61)	1.31 (0.89, 1.91)	0.64 (0.39, 1.07)	0.75 (0.43, 1.29)
**How often do household members go hungry?**						
Sometimes/Often (>2 times/month)	1.00	1.00	1.00	1.00	1.00	1.00
Seldom (1–2 times/month)	0.90 (0.58, 1.39)	0.82 (0.52, 1.31)	1.48 (0.88, 2.50)	1.32 (0.75, 2.29)	1.19 (0.66, 2.12)	1.32 (0.68, 2.54)
Never	0.49 (0.36, 0.66)	0.59 (0.42, 0.82)	1.00 (0.68, 1.46)	1.13 (0.75, 1.70)	0.45 (0.30, 0.69)	0.80 (0.49, 1.32)
**Social support**						
Strong support	1.00	1.00	1.00	1.00	1.00	1.00
Moderate support	0.69 (0.50, 0.95)	0.73 (0.52, 1.02)	0.97 (0.67, 1.40)	1.04 (0.70, 1.54)	0.40 (0.26, 0.62)	0.52 (0.32, 0.84)
Poor support	1.03 (0.70, 1.52)	0.96 (0.64, 1.46)	1.14 (0.72, 1.82)	1.37 (0.84, 2.24)	1.27 (0.78, 2.06)	1.49 (0.84, 2.63)
**Any household members consumed alcohol, past 3 months**						
None	1.00	1.00	1.00	1.00	1.00	1.00
Any	1.92 (1.42, 2.60)	1.80 (1.29, 2.50)	2.32 (1.68, 3.21)	1.95 (1.36, 2.79)	6.24 (4.33, 8.99)	5.74 (3.80, 8.67)
**Recruitment site**						
Inpatient wards	1.00	1.00	1.00	1.00	1.00	1.00
Outpatient clinics	1.06 (0.81, 1.39)	1.09 (0.81, 1.46)	1.00 (0.74, 1.36)	1.07 (0.77, 1.48)	1.25 (0.83, 1.89)	1.63 (1.03, 2.57)
Emergency/casualty wards	0.95 (0.64, 1.41)	0.84 (0.56, 1.26)	1.35 (0.90, 2.03)	1.34 (0.87, 2.08)	0.88 (0.47, 1.62)	1.01 (0.52, 1.98)
**Reasons for HIV testing**						
Just wanted to know/plan for future	1.00	-	1.00	-	1.00	-
AIDS symptoms/concern about current illness	1.63 (1.29, 2.05)		1.39 (1.08, 1.80)		1.67 (1.20, 2.34)	
Other	1.37 (0.82, 2.30)		0.92 (0.48, 1.75)		2.25 (1.18, 4.30)	
**HIV status**						
HIV negative	1.00	1.00	1.00	1.00	1.00	1.00
HIV positive	1.68 (1.36, 2.09)	1.49 (1.17, 1.88)	1.42 (1.12, 1.80)	1.15 (0.88, 1.51)	2.34 (1.70, 3.22)	1.88 (1.30, 2.73)

RRR: Relative risk ratio.

CI: Confidence interval.

### Alcohol use in the year following HCT

**Figure 1:**
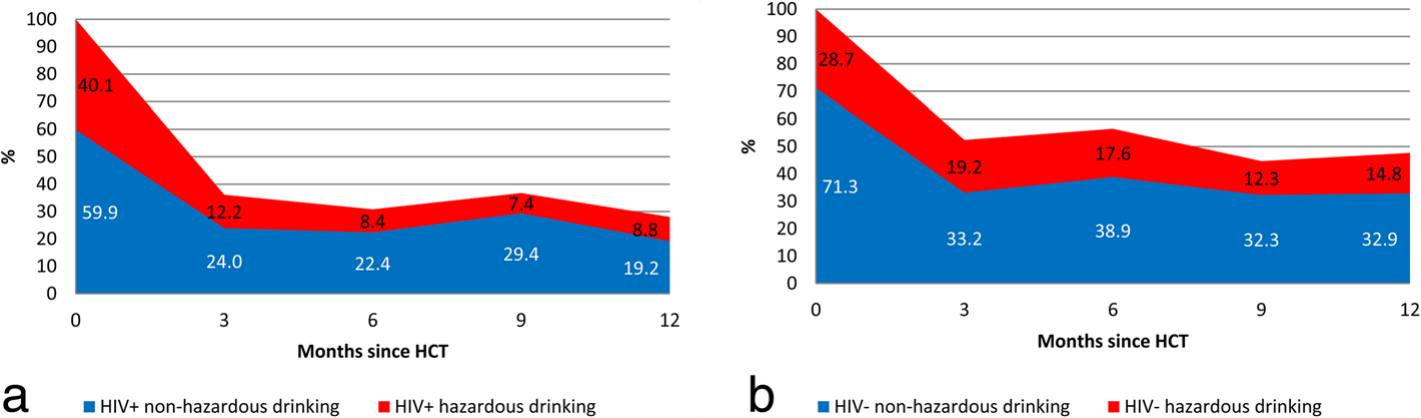
**Hazardous and non-hazardous drinking at study visits following HIV counseling and testing (HCT), among current drinkers at baseline, by HIV status. 1a**. 294 HIV infected drinkers. **1b**. 247 HIV negative drinkers.

Among current drinkers at baseline, both hazardous and non-hazardous alcohol consumption decreased dramatically in the first three months after HCT for those with and without HIV infection (Figure 1). Loss to follow-up between hazardous and non-hazardous drinkers at baseline was similar; overall, 8% of hazardous drinkers and 8% of non-hazardous drinkers had no follow-up interviews. Study interview completion at 3, 6, 9, and 12 months was 89%, 85%, 86% and 86% respectively, for those categorized as non-hazardous drinkers at baseline, and 89%, 86%, 84% and 85% respectively, for those categorized as hazardous drinkers at baseline.

Among current drinkers at baseline, independent predictors (p < 0.05) of current, non-hazardous drinking compared to no drinking in the year following HCT were: sex (male), hazardous alcohol use at baseline, and HIV status (negative) (Table 3). These variables were also independent predictors of current, hazardous drinking compared to no drinking, in addition to follow-up month (less hazardous drinking at 9 months). HCT study arm was not associated with non-hazardous or hazardous drinking during follow-up.

**Table 3:** Multinomial logistic regression of non-hazardous and hazardous drinking compared to no current drinking in the year following HCT, among baseline drinkers

	**Non-hazardous drinking RRR (95% CI)**		**Hazardous drinking RRR (95% CI)**	
	**Bivariable**	**Multivariable**	**Bivariable**	**Multivariable**
**Gender**				
Female	1.00	1.00	1.00	1.00
Male	1.95 (1.50, 2.53)	1.81 (1.39, 2.35)	2.07 (1.43, 2.99)	1.83 (1.26, 2.65)
**Age (years)**				
18–25	1.00	-	1.00	-
26–35	0.94 (0.67, 1.32)		0.94 (0.59, 1.51)	
>35	1.15 (0.80, 1.66)		1.11 (0.67, 1.84)	
**Education**				
Primary education or less	1.00	-	1.00	-
More than primary education	0.97 (0.75, 1.26)		1.37 (0.95, 1.95)	
**Occupation**				
Laborer	1.00	-	1.00	-
Business/sales/technical	0.91 (0.68, 1.22)		1.37 (0.89, 2.10)	
Other	0.94 (0.66, 1.33)		1.32 (0.78, 2.25)	
**Religion**				
Protestant	1.00	-	1.00	-
Catholic	0.96 (0.73, 1.27)		1.00 (0.68, 1.47)	
Moslem	0.84 (0.52, 1.38)		0.89 (0.46, 1.74)	
Saved/Pentecostal/Other	0.58 (0.28, 1.21)		0.82 (0.36, 1.90)	
**Marital status**				
Married	1.00	-	1.00	-
Previously married	0.69 (0.51, 0.92)		0.69 (0.45, 1.04)	
Never married	0.84 (0.59, 1.20)		1.04 (0.65, 1.66)	
**Household assets**				
Low	1.00	-	1.00	-
Medium	0.92 (0.70, 1.22)		1.07 (0.73, 1.57)	
High	1.09 (0.72, 1.65)		1.20 (0.68, 2.13)	
**How often do household members go hungry?**				
Sometimes/Often (>2 times/month)	1.00	-	1.00	-
Seldom (1–2 times/month)	0.93 (0.56, 1.54)		1.24 (0.61, 2.50)	
Never	0.97 (0.66, 1.41)		1.13 (0.66, 1.93)	
**Social support**				
Strong support	1.00	-	1.00	-
Moderate support	0.86 (0.60, 1.23)		0.89 (0.52, 1.52)	
Poor support	0.96 (0.63, 1.48)		1.03 (0.56, 1.89)	
**Hazardous alcohol use at baseline?**				
No	1.00	1.00	1.00	1.00
Yes	1.60 (1.21, 2.12)	1.72 (1.29, 2.29)	2.40 (1.65, 3.51)	2.68 (1.81, 3.96)
**Any household members consumed alcohol, past 3 months**				
None	1.00	-	1.00	-
Any	1.22 (0.91, 1.64)		1.48 (1.01, 2.16)	
**Recruitment site**				
Inpatient wards	1.00	-	1.00	-
Outpatient clinics	1.02 (0.71, 1.46)		0.90 (0.56, 1.44)	
Emergency/casualty wards	0.67 (0.41, 1.10)		0.41 (0.20, 0.83)	
**Reasons for HIV testing**				
Just wanted to know/plan for future	1.00	-	1.00	-
AIDS symptoms/concern about current illness	0.85 (0.64, 1.12)		0.88 (0.59, 1.32)	
Other	1.06 (0.62, 1.82)		1.44 (0.64, 3.26)	
**HIV status**				
HIV negative	1.00	1.00	1.00	1.00
HIV positive	0.52 (0.40, 0.68)	0.49 (0.37, 0.65)	0.45 (0.31, 0.65)	0.40 (0.27, 0.58)
**HCT arm**				
Abbreviated HCT	1.00	-	1.00	-
Traditional HCT	1.01 (0.77, 1.31)		0.86 (0.60, 1.23)	
**Follow-up month**				
3 months	1.00	1.00	1.00	1.00
6 months	1.06 (0.83, 1.34)	1.05 (0.82, 1.35)	0.81 (0.59, 1.10)	0.80 (0.58, 1.11)
9 months	1.08 (0.84, 1.39)	1.07 (0.83, 1.39)	0.63 (0.44, 0.90)	0.62 (0.43, 0.90)
12 months	0.80 (0.62, 1.04)	0.79 (0.60, 1.03)	0.74 (0.53, 1.03)	0.72 (0.51, 1.02)

RRR: Relative risk ratio.

CI: Confidence interval.

In multivariable regression limited to current drinkers at baseline with HIV (Table 4), independent predictors of current, non-hazardous alcohol use after HCT were: sex (male) and ART status (on ART more likely to not drink). Independent predictors of current, hazardous alcohol consumption after HCT among those with HIV were: hazardous alcohol use at baseline, and recruitment at an emergency/casualty ward (less hazardous drinking compared to those recruited at an inpatient ward). Linkage to care and HCT study arms were not associated with non-hazardous or hazardous drinking during follow-up.

**Table 4:** Multinomial logistic regression of non-hazardous and hazardous drinking compared to no current drinking in the year following HCT, among HIV infected baseline drinkers

	**Current, non-hazardous drinking RRR (95% CI)**		**Current, hazardous drinking RRR (95% CI)**	
	**Bivariable**	**Multivariable**	**Bivariable**	**Multivariable**
**Gender**				
Female	1.00	1.00	1.00	1.00
Male	1.93 (1.32, 2.82)	1.92 (1.31, 2.82)	1.65 (0.96, 2.86)	1.42 (0.78, 2.56)
**Age (years)**				
18–25	1.00	-	1.00	-
26–35	1.00 (0.62, 1.59)		0.76 (0.38, 1.55)	
>35	1.21 (0.70, 2.08)		0.80 (0.35, 1.81)	
**Education**				
Primary education or less	1.00	-	1.00	-
More than primary education	1.17 (0.81, 1.71)		1.25 (0.72, 2.16)	
**Occupation**				
Laborer	1.00	-	1.00	-
Business/sales/technical	0.72 (0.47, 1.10)		0.96 (0.53, 1.74)	
Other	0.86 (0.51, 1.46)		1.14 (0.55, 2.35)	
**Religion**				
Protestant	1.00	-	1.00	-
Catholic	0.87 (0.58, 1.29)		0.83 (0.47, 1.45)	
Moslem	0.66 (0.32, 1.34)		0.46 (0.15, 1.48)	
Saved/Pentecostal/Other	0.49 (0.18, 1.37)		0.86 (0.24, 3.01)	
**Marital status**				
Married	1.00	-	1.00	-
Previously married	0.79 (0.53, 1.17)		0.92 (0.51, 1.64)	
Never married	0.83 (0.46, 1.51)		1.51 (0.70, 3.24)	
**Household assets**				
Low	1.00	-	1.00	-
Medium	1.04 (0.69, 1.56)		1.20 (0.67, 2.15)	
High	0.91 (0.50, 1.64)		1.60 (0.75, 3.43)	
**How often do household members go hungry?**				
Sometimes/Often (>2 times/month)	1.00	-	1.00	-
Seldom (1–2 times/month)	0.76 (0.39, 1.49)		1.00 (0.41, 2.46)	
Never	0.81 (0.50, 1.32)		0.85 (0.43, 1.69)	
**Social support**				
Strong support	1.00	-	1.00	-
Moderate support	0.59 (0.36, 0.96)		0.58 (0.28, 1.19)	
Poor support	0.69 (0.38, 1.25)		0.90 (0.41, 1.99)	
**Hazardous alcohol use at baseline?**				
No	1.00	1.00	1.00	1.00
Yes	1.49 (0.96, 2.31)	1.42 (0.91, 2.23)	3.21 (1.79, 5.75)	3.15 (1.74, 5.70)
**Any household members consumed alcohol, past 3 months**				
None	1.00	-	1.00	-
Any	1.04 (0.68, 1.59)		1.52 (0.85, 2.72)	
**Recruitment site**				
Inpatient wards	1.00	1.00	1.00	1.00
Outpatient clinics	1.04 (0.62, 1.75)	1.09 (0.65, 1.83)	0.70 (0.34, 1.42)	0.65 (0.30, 1.41)
Emergency/casualty wards	0.52 (0.27, 1.01)	0.57 (0.29, 1.13)	0.24 (0.08, 0.72)	0.26 (0.08, 0.85)
**Reasons for HIV testing**				
Just wanted to know/plan for future	1.00	-	1.00	-
AIDS symptoms/concern about current illness	0.84 (0.57, 1.24)		1.40 (0.80, 2.46)	
Other	1.50 (0.64, 3.49)		2.71 (0.93, 7.92)	
**HCT arm**				
Abbreviated HCT	1.00	-	1.00	-
Traditional HCT	0.96 (0.66, 1.41)		1.01 (0.59, 1.72)	
**Linkage to care arm**				
Enhanced linkage to care	1.00	-	1.00	-
Standard linkage to care	0.97 (0.67, 1.42)		0.98 (0.57, 1.67)	
**Follow-up month**				
3 months	1.00	1.00	1.00	1.00
6 months	0.83 (0.56, 1.21)	0.89 (0.60, 1.33)	0.65 (0.40, 1.08)	0.68 (0.40, 1.16)
9 months	1.27 (0.88, 1.84)	1.48 (1.00, 2.22)	0.75 (0.42, 1.36)	0.84 (0.45, 1.57)
12 months	0.69 (0.47, 1.01)	0.83 (0.54, 1.26)	0.78 (0.48, 1.26)	0.88 (0.52, 1.49)
**Baseline CD4 count**				
<200	1.00	-	1.00	-
200–349	1.59 (0.94, 2.68)		1.15 (0.53, 2.47)	
350–499	1.81 (1.03, 3.18)		1.50 (0.68, 3.28)	
> = 500	1.60 (0.95, 2.71)		1.47 (0.68, 3.19)	
**Taking ART?**				
No	1.00	1.00	1.00	1.00
Yes	0.52 (0.36, 0.76)	0.50 (0.33, 0.76)	0.68 (0.38, 1.21)	0.61 (0.33, 1.13)

RRR: Relative risk ratio.

CI: Confidence interval.

## Discussion

In a large sample of persons receiving HCT in Uganda, almost one-third reported current alcohol use, and one-third of those met criteria for hazardous alcohol consumption. Among current drinkers, both hazardous and non-hazardous drinking declined dramatically in the first three months after HCT, and the decline was more dramatic among those with HIV. In the year following HCT, continued drinking at both hazardous and non-hazardous levels, as compared to abstinence, was more likely among men, and those who were not infected with HIV. Among those with HIV, current non-hazardous drinking was less likely among those on ART.

Our findings on lifetime and current alcohol consumption are consistent with those of prior studies of alcohol use in SSA. Alcohol use has previously been reported to be less common among women in Uganda [1], and alcohol use is prohibited in the Moslem and Evangelical religions. Persons with HIV in our study were more likely to have ever consumed alcohol (HIV status was associated with past and current hazardous use), which is consistent with the increasing evidence of a link between alcohol consumption and HIV infection [6]. We additionally found that household alcohol use was associated with current hazardous and non-hazardous alcohol use at baseline, with increased risk for hazardous use. This suggests that peer norms play a role in alcohol consumption, as has been shown among adolescents and college students [37]. In a previous study conducted in Uganda, less loneliness and higher levels of social interactions were associated with an increased frequency of alcohol use, suggesting that alcohol consumption is socially normative [38].

The decline in self-reported alcohol consumption during follow-up is consistent with our previous finding of high levels of self-reported abstinence just prior to and immediately following the start of ART in a cohort of persons initiating ART in rural southwest Uganda [25]. The current findings extend the literature by examining a key period in the time course of HIV infection, that is, prior to HCT and in the year immediately following HCT. Self-reported alcohol consumption declined most dramatically in the first three months following HCT, and additional declines occurred among those with HIV, at least among non-hazardous drinkers, when ART was initiated.

There are several plausible explanations for the decreases in alcohol consumption. First, alcohol consumption may have declined as a result of the precipitating factors that led the participants to seek health care services at Mulago Hospital. In our previous study, those with lower health scores were more likely to become abstinent [25], and health conditions have been previously cited as a reason for attempts to reduce or abstain from alcohol use [39-41]. Those seeking health care may represent a select group, i.e. they may be those who are currently ready and willing to engage in health-preserving behavior in general, or may be those who are too ill to drink. Perhaps the health condition serves as a “learnable moment”, in which the patient recognizes a link between their health and drinking, which spurs reductions in drinking independent of any intervention [42]. In addition, contact with health care providers may be an intervention in itself. In a study of patients in residential drug and alcohol detoxification programs, receipt of primary care was associated with a significant decrease in alcohol use severity [43]. The counseling included during the HCT provided by this study may also have been an intervention, although neither of the two counseling protocols (traditional or abbreviated) explicitly addressed alcohol use. However, there were no differences in alcohol use after HCT by method of counseling, suggesting no effect of HCT counseling. The majority (80%) of those who were infected with HIV received follow-up care [30], and the ISS clinic at Mulago Hospital discourages all alcohol consumption, especially at ART initiation. In addition, men who consume more than 14 drinks per week and women who consume more than 11 drinks per week, and lighter drinkers who do not quit drinking, are referred to a psychiatrist for counseling. This may explain the greater decrease in drinking among those with HIV, and especially among patients on ART. ART initiation may have a variety of health benefits beyond the direct effects on HIV disease. It has been found to be associated with other positive health outcomes, such as reduced internalized stigma [44], increased food security, nutritional status, physical health status [45], and decreased depression status [46]. If alcohol consumption declined for the reasons noted above, then these findings suggest that hospital and clinic entry, HCT, and for those infected with HIV, ART initiation, may be opportune times to intervene on alcohol use, and future interventions should capitalize on these opportunities. Just as emergency department visits for alcohol-related trauma provide a “learnable moment” for alcohol reduction, HCT may also provide such an opportunity.

However, it is also possible that the reductions in alcohol use are due to assessment reactivity. Assessment reactivity occurs when the assessment of alcohol consumption itself increases self-awareness of alcohol problems and thus triggers reductions in alcohol consumption [47]. A related explanation could be the Hawthorne effect, in which those being studied change their behavior. Either of these effects could explain the trends towards decreasing alcohol consumption seen in other prospective research studies, including a study in women with HIV and a study of injecting drug users in the United States [26,48]; however, they do not explain why the decreases were greater in those with HIV versus those without. Another reason for the decline in drinking may be regression to the mean, whereby by selecting the current drinkers at baseline, some revert to non-drinking on subsequent visits. However, one would expect an equal fraction of non-drinkers to become drinkers, and this was not the case (only 13% of non-drinkers at baseline reported any drinking at follow-up).

### Limitations

The most important limitation is that the measures of drinking are self-reported, and under-report is a reasonable concern. Socially desirable reporting of stigmatized behavior is a common problem in health care settings. Those with HIV were likely to have been instructed to cease drinking during their HIV clinic visits, and might fear that ART may be denied if they report drinking [49]. We have previously reported on the use of biomarkers to detect under-reported alcohol use in Uganda [50,51]; however, we were unable to collect biological markers to corroborate self-report in this study. Nonetheless, the correlates of baseline and follow-up drinking were consistent with previous literature; for example, men drinking and Moslems not drinking, which alleviates some of this concern. Another limitation is that the study was not designed to examine alcohol consumption; therefore, we were unable to explore psycho-social correlates of drinking after HCT. An additional limitation is that we did not collect data on drug use, and thus are unable to comment on any changes in drug use that may have occurred following HCT. However, drug use in Uganda is uncommon; as reported by the World Health Organisation, <1% of men and women in Uganda in 2004 had a drug use disorder [52]. Similarly, in a study of HIV-positive and HIV-negative female sex workers in Kampala, a group one would suspect would have high levels of drug use, only 8% reported ever using marijuana or khat, and only 2 women (0.2%) had ever injected heroin, while 78% of women reported alcohol use [53]. Therefore, it is likely that very few of the participants were illicit drug users. Another limitation is that our results may not be generalizable to other populations in SSA. For example, a study in South Africa of HIV-infected patients on ART found a higher proportion of hazardous drinking among ever drinkers (75% of men and 55% of women, as defined using AUDIT cutoffs of ≥8 for men and ≥6 for women) than was observed in our study [54]. A population-based study in Botswana, defining heavy drinking as >14 drinks/week for women and >21 drinks/week for men, also found a higher proportion of heavy drinking (54% of men and 58% of women) than among drinkers in our study [55]. Social controls may be stronger in Uganda than some SSA countries; HIV patients in particular in Uganda may be more compliant to the advice they receive from health care workers than patients in other countries. Thus, our results should be interpreted with caution when applied to other settings. Further study of how alcohol consumption changes after HCT in other countries in SSA would be valuable.

## Conclusions

Despite these limitations, our findings significantly extend the previous literature of alcohol use in Uganda and SSA, to examine patterns in self-reported alcohol consumption prior to and after HIV testing. The findings suggest that HIV testing and ART initiation may be ideal venues for brief interventions. Screening and brief intervention (SBI) have been found to be efficacious in reducing alcohol consumption among those in primary care [56]; however, it is unknown whether such findings translate to resource-constrained settings [56]. Further work is needed to determine whether such interventions will be efficacious and whether they may be feasible to implement in SSA.

## Abbreviations

AUDIT-C: Alcohol Use Disorders Identification Test - Consumption; ART: Antiretroviral therapy; CI: Confidence interval; HCT: HIV counseling and testing; RRR: Relative risk ratio; SBI: Screening and brief intervention; SSA: Sub-Saharan Africa.

## Competing interests

The authors declare that they have no competing interests.

## Authors’ contributions

TC, DB, MK, RW, and JH designed the study. JH and RF conducted the statistical analyses. JH drafted the manuscript, with input from all authors. SB, RW, and RF led the acquisition of the data. All authors read and approved the final manuscript.

## Pre-publication history

The pre-publication history for this paper can be accessed here:

http://www.biomedcentral.com/1471-2334/14/403/prepub
